# Observations and Experiments on the Action of Certain Anæsthetics, Alone or Mixed, on the Fibres of the Fifth Pair of Nerves

**Published:** 1887-07

**Authors:** Laurence Turnbull, E. P. Reichert, John D. Thomas

**Affiliations:** Philadelphia, Pa.; Philadelphia, Pa.; Philadelphia, Pa.


ARTICLE IX.

### OBSERVATIONS AND EXPERIMENTS ON THE ACTION OF CERTAIN ANÆSTHETICS, ALONE OR MIXED, ON THE FIBRES OF THE FIFTH PAIR OF NERVES.

---

BY DRS. LAURENCE TURNBULL, E. P. REICHERT AND JOHN D. THOMAS, PHILADELPHIA, PA,

---

Dr. Reichert, at my suggestion, was so kind as to perform with us the following experiments in regard to the action of certain anæsthetics when the fifth pair of nerves were irritated. The following was the Doctor's reply to my letter of April 27, 1885:

"In regard to your inquiry about Lauder Brunton's experiments on the reflex inhibition of the heart, I may say, that I have read his paper but do not remember where it was published. We can cause a reflex inhibition of the heart, by irritating any sensory nerve by any of the ordinary means of irritation. I have found in some of my experiments (not yet published), that you can produce a greater degree of inhibition by irritating certain sensory nerves than by irritating the trunk of the vagus itself, when the same degree of irritation is used in both cases. The easiest way to demonstrate the power of the branches of the fifth nerve, when irritated to cause inhibition, is to anæsthetize a rabbit and place some strong aqua ammonia to the nose. This acts as a powerful irritant to nasal fibres of the fifth, and brings about reflex inhibition—complete stoppage if necessary."

Dr. Reichert took a large sized rabbit and placed it cautiously, but with some difficulty, under the influence of a mixture of ether and bromide of ethyl ℥ ii to Oi; requiring a considerable amount to bring it fully under its influence. The trachea was opened and a tube introduced. The carotid artery was opened, cut and ligatured and a tube introduced which was filled with soda solution, free from air bubbles, and connected with Ludwig's Kymographion. A cylinder with blackened paper was set in motion by clock-work, and a delicate pen of thin platinum foil was made to record every second by means of an electro-magnet. Dr. R. then caused reflex inhibition of the heart, by irritation of the nasal branch of the fifth pair of nerves, by touching the nostrils with a strong solution of aqua ammonia, when chloroform was applied. The animal was allowed to recover. Chloroform was then placed upon a sponge and applied to the tube only. In an instant the rabbit was dead, showing the much more dangerous character of that agent as an anæsthetic. An examination of the organs of the animal was then made, by removing the sternum and ribs, when it was found that death had taken place from the stoppage of the heart's action, the lungs were found perfectly normal as well as all the organs excepting the liver, which was badly tuberculous, a condition frequently found in the rabbit. A perceptible heart motion continued for some time after death, but with insufficient force to propel the blood, the indicator remaining perfectly quiet.

A dog was then anæsthetized, but required a much smaller amount of the ether mixture than the rabbit, and the irritation applied as before. The same experiment was performed upon it, but the action on the heart was not as prompt. After a short time, the dog was to all intents dead, from too large a quantity of the mixture, but by active compression and relaxation of the chest walls by the hands of the operator, it was resuscitated. Then the dog was placed fully under a solution of chloral by the vein, the superior laryngeal nerve was dissected, and an induction current was applied. The effect upon the heart was instantaneous, to such a degree as to cause complete suspension of its pulsation. The result was the same when the current was applied directly to the pneumo gastric.

A rabbit was then placed under the anæsthetic influence of ether, to prepare him for the experiment; he was then allowed to recover. The arterial pressure and pulse were then recorded, aqua ammonia was applied to his nostrils, with a result similar to that observed in the first experiment. There was administered a combination of nitrous oxide gas and chloroform, by means of the chloroform mixer of the Buffalo Dental Manufacturing Company. The chloroform is confined in a receptacle below the gas passage, which is closed by a screw-valve. A stem, covered by a fibrous sheath, extends downwards from the valve into the chloroform. When the mixture was made, the valve was loosened by the wheel handle and drawn upwards, raising with it the covered stem which brought with it a certain amount of chloroform into the gas passage, where it was exposed to the current of gas as it passed to the inhaler from the bag. A small piston closes the passage to the chloroform reservoir, as the handle is raised, cutting off the escape of vapor. With this apparatus the mixture may be made or withheld, and more or less chloroform be given, as the judgment of the operator may dictate. The results showed the advantage of using a full supply of the nitro-oxide gas, and not a partial anæsthesia, which is a most unsafe plan, as the apparatus is not designed to save the quantity of gas administered.

The last experiment made was allowing the animal fully to recover, from the mixed anæsthetic, and placing it under the full effects of Squibbs' stronger ether for anæsthesia, which is now so much employed in surgery. The ether was crowded upon it until the *respiration and heart had almost ceased to beat.* Then was introduced into it a solution of 100th of a grain of sulphate of atropia, to see if it had any power to stimulate the heart or restore respiration, but it produced no such results, and the animal was dead.

OBSERVATIONS BY DR. TURNBULL.

The more frequent employment by surgeons of Squibbs' ether, or what is known as Ether Fortior U. S. P., the stronger ether—a liquid composed of about 94 per cent. of ethyl oxide and about 6 per cent. of alcohol, and only containing a little water—brings this anæsthetic near to the character of chloroform. This more powerful agent than the ordinary ether has certain advantages in the rapidity with which true anæsthesia is produced, but as a matter of course enhances the dangers attending its use in a certain class of individuals, as the aged, the inebriate and the very young. If the same amount of caution is employed in the use of this more powerful agent as in the use of chloroform, the number of deaths would be less. It must be always borne in mind that in full anæsthesia, no matter what agent is employed, there is a suspension of the life forces, and but a step to death. The administration of morphia subcutaneously will increase the risk, as there are many individuals who have a certain idiosyncrasy, and cannot bear even what is known as a small dose of morphia without great disturbance of the stomach, or faintness. It has been suggested to add to it atropine, but our experiments, I think, demonstrate that it will not relieve the heart when fully under the influence of the stronger ether. It is also true that small doses of the morphine are now recommended, but our experience has fully proven that when ether is employed, there are no agents which relieve the irritation of the broncho-pulmonary mucous membrane so well as keeping the skin warm and free from moisture or draughts of cool air, and now and then dipping the sponge in hot water, and getting rid of cold water, and even ice which forms on the inhaler or sponge. Another good plan is to keep the bottle of ether in warm water (not hot.) Above all, no one should give the ether who has not had some practical experience, and is not desirous of witnessing the operation; let the whole attention be given to the patient and never crowd it after the patient has become fully anæsthetized. Keep the influence of the ether by short and slight inhalations, well diluted with warm air. In the use of chloroform it should never be employed unless to the very aged, the hard drinker or young children, or in midwifery or gynæcological operation, when the patient can be placed in the horizontal position. There are a few rare cases, where, after a most careful and conscientious use of ether, the patient will not come promptly under its anæsthetic influence; then it will be proper to use hydrobromic ether or chloroform.

We consider all mixed anæsthetics more dangerous than one alone, but the physician should have a full knowledge of the constitution of the individual. There is not quite so much risk to life with chloroform as with ether if morphine is employed subcutaneously, but owing to the same reasons expressed under ether, I do not recommend it, and I know of but few surgeons, either in this country or in Europe, who resort to its use.—*Dental Advertiser.*

